# Cell therapy in Sjögren's syndrome: opportunities and challenges

**DOI:** 10.1017/erm.2024.21

**Published:** 2024-10-23

**Authors:** Yangyang Lu, Rongjing Shi, Wenqin He, Qi An, Jingwen Zhao, Xinnan Gao, Baiyan Zhang, Liyun Zhang, Ke Xu, Dan Ma

**Affiliations:** 1 Third Hospital of Shanxi Medical University, Shanxi Bethune Hospital, Shanxi Academy of Medical Sciences, Tongji Shanxi Hospital, Taiyuan, China; 2 Shanxi Province Clinical Research Center for Dermatologic and Immunologic Diseases (Rheumatic diseases), Taiyuan, China; 3 Shanxi Province Clinical Theranostics Technology Innovation Center for Immunologic and Rheumatic Diseases, Taiyuan, China; 4 Shanxi Academy of Advanced Research and Innovation, Taiyuan, China

**Keywords:** CAT-T, cell therapy, extracellular vesicles, HSCs, iPSCs, MSCs, Sjögren's syndrome

## Abstract

Sjögren's syndrome (SS) is a chronic autoimmune disease caused by immune system disorders. The main clinical manifestations of SS are dry mouth and eyes caused by the destruction of exocrine glands, such as the salivary and lacrimal glands, and systemic manifestations, such as interstitial pneumonia, interstitial nephritis and vasculitis. The pathogenesis of this condition is complex. However, this has not been fully elucidated. Treatment mainly consists of glucocorticoids, disease-modifying antirheumatic drugs and biological agents, which can only control inflammation but not repair the tissue. Therefore, identifying methods to regulate immune disorders and repair damaged tissues is imperative. Cell therapy involves the transplantation of autologous or allogeneic normal or bioengineered cells into the body of a patient to replace damaged cells or achieve a stronger immunomodulatory capacity to cure diseases, mainly including stem cell therapy and immune cell therapy. Cell therapy can reduce inflammation, relieve symptoms and promote tissue repair and regeneration of exocrine glands such as the salivary glands. It has broad application prospects and may become a new treatment strategy for patients with SS. However, there are various challenges in cell preparation, culture, storage and transportation. This article reviews the research status and prospects of cell therapies for SS.

## Introduction

Sjögren's syndrome (SS) is a chronic autoimmune disease caused by immune system disorders. Its prevalence is 60.82/100 000 people per year, and the average male-to-female ratio is 1:9 (Refs 1, 2). The main clinical manifestations of SS are dry mouth and eyes caused by the destruction of exocrine glands, such as the salivary and lacrimal glands. Approximately 30–40% of patients also have systemic manifestations, including synovitis, interstitial pneumonia, interstitial nephritis and vasculitis (Refs 1, 3). It is a unique disease known as primary Sjögren's syndrome (pSS), and when it is secondary to other autoimmune diseases such as rheumatoid arthritis (RA), systemic lupus erythematosus (SLE) and systemic sclerosis (SSc), it is called secondary Sjögren's syndrome) (Ref. 2). The risk of lymphoma in patients with pSS is approximately 5–10%. The main types are low-grade B-cell non-Hodgkin lymphoma and marginal tissue band types, such as mucosa-associated lymphoid tissue lymphoma (MALT) (Refs 1, 4).

**Figure 1.**
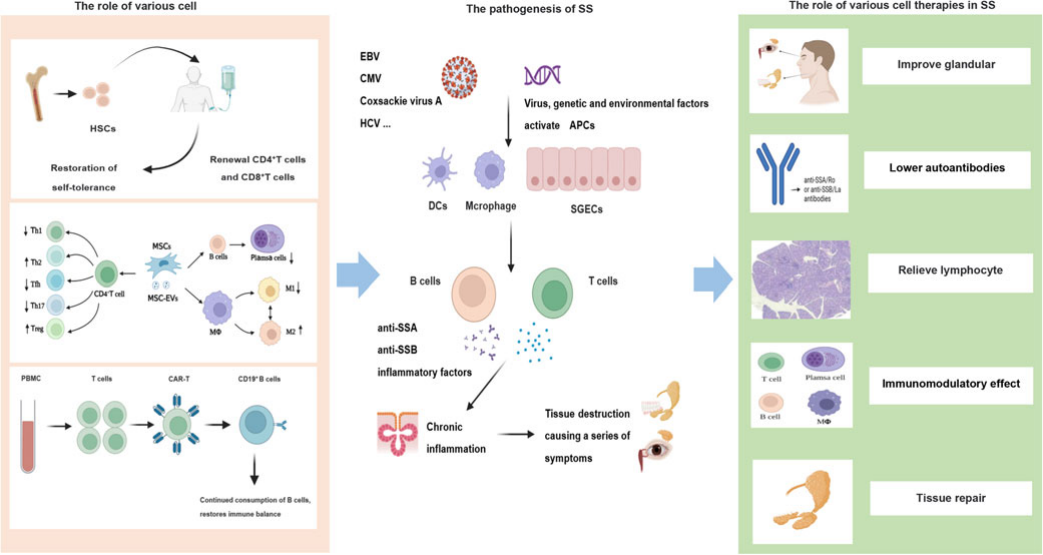
Pathogenesis and cell therapy of SS. HSCs, haematopoietic stem cells; MSCs, mesenchymal stem cells; MSC-EVs, EVs released by MSCs; CAR-T, chimeric antigen receptor T cell; SGECs, salivary gland epithelial cells.

The pathogenesis of SS is complex and has not been fully elucidated (Fig. 1). Known factors include environmental, genetic and infection factors, and cases are characterised by over-activation of innate and adaptive immunity (Refs 1, 2, 5, 6). The interaction between genetic susceptibility and environmental factors, especially after infection with Epstein–Barr virus, cytomegalovirus, coxsackie virus A and enterovirus, can activate antigen-presenting cells (APCs) such as mononuclear macrophages, dendritic cells (DCs) and salivary gland epithelial cells (SGECs) (Refs 2, 5, 7, 8). APCs have been shown to interact with T and B cells and stimulate the activation of T and B cells, resulting in T cells differentiating into different cell subsets and the secretion of a large number of inflammatory factors. The T cells can further activate B cells. While secreting a large number of inflammatory factors, B cells can differentiate into plasma cells to produce immunoglobulins (Igs) and autoantibodies (anti-SSA and anti-SSB antibodies, respectively), thereby causing tissue destruction and a series of various clinical symptoms (Ref. 5). Meanwhile, B cells are surrounded by T cells to form an ectopic germinal centre, which increases the risk of lymphoma in patients with pSS (Refs 5, 7).

Currently, there are no approved drugs for the treatment of pSS, mainly for symptomatic treatment of dry mouth and eyes. Glucocorticoids and disease-modifying antirheumatic drugs are used when systemic involvement occurs (Refs 7, 9). There are also some biological agents, such as B-cell-targeted therapy drugs, including rituximab, belimumab, epratuzumab and baminercept. T cell-targeting drugs include abatacept and alefacept, and cytokine-targeting drugs include infliximab and tofacitinib (Refs 7, 10, 11). Long-term use of glucocorticoids in traditional medicine can cause metabolic abnormalities, such as osteoporosis, hypertension, hyperglycaemia and central obesity, and may also lead to femoral head necrosis and infection. DMARDs have poor curative effects in some patients, have a long onset time and their long-term use increases the risk of infection. Biologics are fast-acting and have potent activity. Currently, biologic agents treat SS mainly in B cells. For example, multiple clinical trials of rituximab for SS have inconsistent results (Refs 12, 13, 14). Belimumab and ianalumab clinical studies have found that they can reduce the ESSDAI score and improve dryness, but there may be local injections of reaction to the wound to increase the chance of infection (Refs 13, 14); therefore, we still need to continue to explore the effectiveness of biological treatment and security. However, these treatments can only control inflammation, and they do not repair tissue damage. Therefore, identifying methods to regulate immune disorders while repairing damaged tissues are imperative.

Cell therapy involves the transplantation of autologous or allogeneic normal or bioengineered cells into the body of a patient to replace damaged cells or achieve a stronger immunomodulatory capacity to cure diseases (Ref. 15). Cell therapy, a form of regenerative medicine that aims to repair damaged cells by reducing inflammation and regulating the immune system, has been widely used in autoimmune and blood system diseases and is gradually becoming a novel therapeutic approach. Cell therapies can be divided into stem- and immune cell therapies according to the cell type, showing prospective applications for various diseases. We have searched for articles related to this topic in the past 20 years through PubMed, CNKI, Wanfang, and other websites in order to review the research progress and prospects of cell therapy in SS (Fig. 1, Table 1).

**Table 1.** Research on cell therapy in SS

Cell type	Cell origin	Animal/patient	Results	References
HSCs	Auto-SCT	pSS complicated with interstitial lung disease	Dry eyes, shortness of breath, ESR, CRP, number and size of pulmonary nodules improved	Ref. 19
		SS patients (Clinical Trials)	Clinical symptoms continuously relieved, the overall survival rate was 90%, the progression-free survival rate was 70%	Ref. 19
		SS complicated with MM	No change of SS (no progression MM)	Ref. 20
			No improvement of SS (Relapse of MM, died)	Ref. 21
		SS complicated with MALT	Dryness and salivary gland function not improved, continuous deterioration of salivary gland function, mild salivary gland fibrosis, lymphoma not relapse	Ref. 22
		SS complicated with immunoblastic lymphoma	No improvement of SS (died because of *P. carnii* pneumonia)	Ref. 23
MSCs	BMMSCs	NOD mice	The salivary flow rate, and lymphocyte infiltration in the gland improved, the number of Th17 and Tfh cells decreased, Th2 and Treg cells increased	Ref. 26
	UCMSCs	NOD mice	The salivary flow rate, and lymphocyte infiltration in the gland improved, the number of Th17 and Tfh cells decreased, Treg cells increased, the production of IL-2, IL-6, TNF-*α*, IFN-*γ* downregulate, IL-10, TGF-*β* upregulate	Refs 29, 30, 31
	LGMSCs	NOD mice		Ref. 32
	UCMSCs	pSS patients (Clinical Trials) (NCT00953485)	The salivary flow rate, SSDAI and VAS improved, serum antibodies decreased	Ref. 26
			The serum IL-12 level of the patients, and DCs expression of IL-12 mRNA decreased	Ref. 30
	BMMSCs UMMSCs	SS patients (Clinical Trials) (NCT00698191)	No adverse events were observed in the heart, kidneys, gastrointestinal tract, nervous system, or haematological system after MSCs infusion for 1 month, and the malignant tumour was reported in one SS	Ref. 33
	ASCs	SS patients (Clinical Trials) (NCT04615455)	No results posted (phase 2)	
MSC-EVs	LGMSC-Exos	NOD mice	The salivary flow rate improved, the number and area of salivary gland lymphocyte infiltration foci reduced, the number of Th17 cells decreased, Treg cells increased, the expression of IL-17A, IFN-*γ*, IL-6, and TNF-*α* decreased, TGF-*β* and IL-10 increase	Refs 32, 46
		pSS patients (in vitro)	The proportion of plasma cells decreased	Ref. 46
	UCMSC-Exos	pSS patients (in vitro)	The autophagy level of CD4^+^T cells reduced, the number of Th17 cells decreased, Treg cells increased, the expression of IL-17A, IL-17F, IFN-*γ*, IL-6, and TNF-*α* decreased, TGF-*β* and IL-10 increased	Ref. 47
iPSCs	iPSC-MSCs	NOD mice	Lymphocyte infiltration and serum antibodies decreased, the expression of CD19, CD79a, CD79b, Ighg3, CD4, Fxop3, Icosl, and CD40 downregulated, IL-10 upregulated	Ref. 64
	iPSC-MSC-EVs	NOD mice	Lymphocyte infiltration, serum antibodies and B-cell-related gene expression decreased	Ref. 64
SGSCs	hSGSCs	Radiation-damaged Wistar rats	The body weight of the rats in the hSGSC group, the salivary flow rate increased, and the structure of salivary glands showed a compact acicular structure similar to that of the undamaged normal rats	Ref. 72
Immune cell	CD19/BCMA chimeric antigen receptor T cells	Patients with refractory SS (Clinical Trials) (NCT05085431)	No results posted (early phase 1)	

HSCs, haematopoietic stem cells; auto-SCT, autologous stem cell transplantation; pSS, primary Sjögren's syndrome; ESR, erythrocyte sedimentation rate; CRP, C-reactive protein; MM, multiple myeloma; MALT, mucosa-associated lymphoid tissue lymphoma; MSCs, mesenchymal stem cells; BMMSCs, bone marrow-derived mesenchymal stem cells; IL, interleukin; IFN, interferon; TNF, tumour necrosis factor; TGF, transforming growth factor; UCMSCs, umbilical cord-derived mesenchymal stem cells; LGMSCs, labial gland-derived mesenchymal stem cells; ASCs, allogeneic adipose-derived mesenchymal stem cells; SSDAI, SS disease activity index; VAS, visual analogue scale; MSC-EVs, EVs released by MSCs; iPSCs, induced pluripotent stem cells; SGSCs, salivary gland stem cells.

## Stem cell therapy

Stem cells can be classified as totipotent, pluripotent or monopotent based on their differentiation potential. Totipotent stem cells, including embryonic stem cells (ESCs), can become totipotent. They maintain their normal karyotype and pluripotency during the process of infinite proliferation and produce whole organisms. However their clinical application is limited owing to ethical concerns. Pluripotent stem cells, including haematopoietic stem cells (HSCs), mesenchymal stem cells (MSCs) and induced pluripotent stem cells (iPSCs) (Ref. 15) can proliferate indefinitely and differentiate into specific tissues but cannot produce whole organisms. Monopotent stem cells are organ-specific stem/progenitor cells, including salivary gland stem cells (SGSCs), skin stem cells and muscle stem cells, which are characterised by their differentiation into one or two closely related cell types. Although their differentiation ability is limited, they play a crucial role in maintaining tissue integrity as progenitor cells that replenish aged and damaged cells (Ref. 16).

### Haematopoietic stem cells

HSCs are primarily found in the bone marrow, peripheral blood and umbilical cord blood, whereas human HSCs are primarily distributed in the bone marrow (approximately 1–5%) compared with its distribution in the peripheral blood (<0.1%). HSC transplantation, which includes autologous HSC transplantation (auto-HSCT) and allogeneic HSC transplantation (allo-HSCT), is a widely used cellular immune therapy (Ref. 17). For auto-HSCT, there are no major histocompatibility complex mismatches. The transplantation process of auto-HSCT is simpler, with fewer transplant-related complications and lower mortality (<1%) than allo-HSCT; however, the disease is prone to relapse (Ref. 17). Allo-HSCT transplantation is a complicated process with complex complications such as immune rejection after the transplantation, among which graft-versus-host disease (GVHD) is the most severe complication (Ref. 17). Auto-HSCT, used for the treatment of autoimmune diseases, eliminates auto-reactive T cells and B cells through radiotherapy and chemotherapy and then infuses the obtained HSCs into the patient, thereby causing CD8^+^T cells and CD4^+^T cells to renew and establish new immune tolerance to reset the immune system (Ref. 18).

SS lung involvement can be manifested as interstitial pneumonia, of which lymphocytic interstitial pneumonia (LIP) is the most common pathological type. One patient with pSS and LIP (Ref. 19) was reported; they had poor results after treatment with methylprednisolone, hydroxychloroquine, methotrexate and pilocarpine. After autologous peripheral blood stem cell transplantation, 18 months of follow-up checks showed that the symptoms of dry eyes and shortness of breath, erythrocyte sedimentation rate, C-reactive protein levels and number and size of pulmonary nodules had significantly improved. Choi *et al*. summarised 101 patients with simple SS treated with autologous stem cell transplantation (auto-SCT) (Ref. 19) and found that clinical symptoms were continuously relieved, with an overall survival rate of 90% and a progression-free survival rate of 70%. Patients with SS complicated by haematological diseases show different results after auto-SCT treatment (Refs 20, 21, 22, 23). In one case of SS with multiple myeloma (MM) (Ref. 20), the myeloma and SS were in complete remission. However, 6 months later, the patient had bilateral parotid and submandibular gland (SMG) enlargement with polyclonal hypergammaglobulinaemia. A SMG biopsy confirmed the recurrence of SS, whereas the myeloma remained in complete remission. In another SS patient with MM and hyperamylasemia (Ref. 21), IgG and amylase levels decreased to normal, and plasma cells decreased in the bone marrow. However, 8 months later, IgG and amylase levels were elevated again, progressive anaemia and dry symptoms were significantly aggravated and the patient died. A patient with SS combined with MALT (Ref. 22) was found to have no recurrence of lymphoma but no improvement in dryness, continuous deterioration of salivary gland function and only mild salivary gland fibrosis after 3 years of follow-up. One patient with SS combined with immunoblastic lymphoma (Ref. 23) had complete remission, whereas the SS relapsed, and the patient died 20 months later because of *Pneumocystis carnii* pneumonia. In patients with pSS complicated by haematological malignant lesions, auto-SCT can improve the disease of the haematological system; however, the improvement in pSS is not apparent.

Before the transplantation of HSCs, different pretreatment schemes should be selected for different patients, such as whole body-radiotherapy or chemotherapy, and chemotherapy drugs, including cyclophosphamide and melphalan. The different schemes have different toxicities and side effects and also have different impacts on patients; therefore, we need to constantly optimise the pretreatment schemes. In addition, infection, recurrence and disease deterioration may occur during the course of treatment; however, pretreatment has certain advantages, such as low immunogenicity, less chance of rejection after transplantation and rebuilding of a new immune system. Further studies are warranted to elucidate the specific mechanisms of immune system rebuilding, which may become a new strategy for treating SS.

### Mesenchymal stem cells

As pluripotent stem cells, MSCs are widely found in various tissues of the body, such as the bone marrow, umbilical cord, fat, placenta, gingiva and skin (Ref. 24). Unlike HSCs, MSCs exhibit high proliferation, differentiation and immune-regulatory abilities. MSCs have a wide range of immunomodulatory functions (Refs 24, 25, 26, 27, 28). The first is their regulation of innate immunity; MSCs can inhibit the activation and maturation of DCs and mononuclear macrophages, induce the transformation of M1 macrophages to M2 macrophages and change the natural killer cell (NK) phenotype. Concomitantly, they weaken antigen presentation and pro-inflammatory function, which may be related to the indirect release of inflammatory factors, such as interleukin (IL)-6, IL-10, indoleamine-2,3-dioxygenase (IDO), nitric oxide (NO), prostaglandin E2 (PGE2), transforming growth factor *β* (TGF-*β*), tumour necrosis factor (TNF)-inducing gene 6 and IL-1 receptor antagonist. In addition, they regulate adaptive immunity: MSCs inhibit the proliferation of T lymphocytes by upregulating the expression of negative cell cycle regulatory protein p27 and the activity of IDO, by producing NO, PGE2, TGF-*β* and hepatocyte growth factors. They inhibit the proliferation of CD4^+^T lymphocytes, downregulate the expression of Thl and Th17 cells and inhibit the secretion of the pro-inflammatory factors interferon-(IFN-)*γ* and IL-17A. They upregulate Th2 and Treg cells, promote the secretion of anti-inflammatory factors IL-4, IL-10 and TGF-*β*, and regulate the balance between Thl/Th2 and Th17/Treg. The proliferation and cytotoxicity of CD8^+^T lymphocytes can be inhibited by upregulating NO synthase. MSCs directly or indirectly release IDO through Th cells in the presence of IFN-*γ*, inhibiting B cell activation, proliferation, plasma cell differentiation, antibody production and chemokine receptor expression.

#### In vivo research of MSCs

In animal experiments, bone marrow-derived MSCs (BMMSCs), umbilical cord-derived MSCs (UCMSCs) and labial gland-derived MSCs (LGMSCs) were used to treat non-obese diabetic (NOD) mice, an SS animal model, and the salivary flow rate of mice was significantly improved, and the number and area of glandular lymphatic infiltrates were reduced (Refs 26, 29, 30, 31, 32). In vitro experiments have shown that BMMSCs (Ref. 26) drive CD4^+^T cells to differentiate into Tregs and Th2 cells, inhibit Th17 and Tfh differentiation and play an immunomodulatory role in improving salivary gland function in mice. UCMSCs (Refs 29, 30, 31) and LGMSCs (Ref. 32) induce Treg cell differentiation, reduce the number of Th17 and Tfh cells, inhibit the production of IL-6, IL-2, TNF-*α*, and IFN-*γ*, promote the production of IL-10 and TGF-*β*, inhibit T cell response and regulate Th17/Treg balance.

In an SS clinical study, Xu *et al*. (Ref. 26) injected UCMSCs into 24 patients with SS. The results showed that the salivary flow rate significantly increased 1 month after UCMSC transplantation, and serum anti-SSA/Ro and anti-SSB/La antibodies significantly decreased. The SS disease activity index and visual analogue scale score improved significantly and were not observed before and after the infusion-related side effects. Shi *et al*. (Ref. 30) injected UCMSCs intravenously (1 × 10^6^/kg body weight) into 10 patients with SS and found that serum IL-12 levels decreased. As DCs mainly secrete IL-12, the IL-12 mRNA also significantly decreased. On the Clinical Trials website, a clinical trial taking place at Copenhagen has been registered; it is a randomised, double-anonymised clinical trial investigating the efficacy of allogeneic adipose-derived mesenchymal stem cells in improving tear fluid. The trial is still ongoing. In a study on the safety of MSCs in the treatment of 404 cases of autoimmune disease (Ref. 33), including 72 patients with SS who failed to respond to conventional treatment and were in the high-activity stage, MSCs were derived from the umbilical cord or bone marrow. Steroids and immunosuppressive drugs were administered during the back transfusion. No adverse events were observed in the heart, kidneys, gastrointestinal tract, nervous system or haematological system after MSC infusion for 1 month. In the follow-up study, five patients developed malignant tumours, namely, two SLE, one RA, one SS and one SSc, and the types of malignant tumours were two lung cancers, two colorectal cancers and one bladder cancer. The appearance of malignant tumours is not an adverse event of MSC transplantation, but it is related to the dysfunction of specific effector cells or decreased secretion of TNF-*α*.

#### In vitro research of MSCs

In addition, MSCs can repair damaged tissues owing to their multidirectional differentiation potential. They differentiate into SGECs, acinar cells and vascular endothelial cells (Ref. 34). MSCs can also improve glandular function through their paracrine effects. Epidermal growth factor (EGF) is a growth factor secreted by the duct cells of salivary glands. It is involved in the growth, regeneration and maintenance of salivary glands and inhibits the apoptosis of salivary epithelial cells. Fibroblast growth factor-2 (FGF-2) promotes the re-epithelialisation of salivary glands. MSCs can effectively repair the salivary glands of NOD mice by increasing the expression levels of EGF and FGF-2 (Ref. 35). Additionally, human BMSCs differentiate into SGECs in a co-culture system, which can express various salivary genes, such as aquaporin 5 (AQP5), E-cadherin and *α*-amylase. A cell structure similar to that of SGECs, such as tight connections and numerous secretory particles, can be observed by electron microscopy (Ref. 24). In conclusion, MSCs have potential applications in repairing SS gland damage and for providing a new treatment method for SS.

Although MSCs have considerable advantages in immune regulation and tissue repair, are widely available, safe and do not cause immune rejection, they have been extensively studied in SS. Nevertheless, many problems remain, such as infection, malignant tumours (Refs 36, 37), pulmonary capillary interception (Refs 38, 39) and ectopic osteogenesis (Ref. 40). In addition, the heterogeneity of MSCs from different sources, optimised preparation process and high cost of storage and transportation still need to be addressed to lay a good foundation for their clinical application in SS therapy.

### Extracellular vesicles derived from MSCs

Studies have shown that MSCs release extracellular vesicles (EVs), either at rest or under stress. They are divided into exosomes (Exos), microparticles (MPs) and apoptotic bodies based on size (Ref. 25). MSC-EVs are lipid bilayer structures that can fuse with the target cell membrane through receptor–ligand interactions or endocytosis to transfer content and play a role in pathophysiological processes (Refs 25, 41, 42). MSC-EVs contain DNA, mRNA and miRNA, lipids, proteins, cytokines, chemokines and growth factors (Refs 25, 42, 43). MSC-EVs have immunomodulatory and tissue repair effects similar to those of MSCs. As the main components of EVs, Exos can simulate the immunoregulatory and tissue repair effects of MSCs, and their immunoregulatory effects are more potent than those of MPs (Ref. 44). It can quickly pass through capillaries when its size is reduced, has stable properties and a strong information transmission ability and does not decline over time. In addition, Exos can prevent problems caused by MSC treatment, such as ectopic osteogenesis, malignant tumour, pulmonary capillary interception and immune rejection.

MSC-EVs exert immunomodulatory effects by delivering miRNAs and regulatory proteins. In this way, they can inhibit the proliferation and activation of T, B, NK and APCs, promote T cell apoptosis, upregulate Treg cells, promote the expression of IL-10 and TGF-*β*, downregulate the expression of Th17 cells and inhibit the expression of IL-17A. They can also induce the transformation of M1 macrophages into M2 macrophages. By delivering mRNA and miRNA, MSC-EVs can activate autophagy and/or inhibit cell apoptosis, necrosis and oxidative stress and upregulate EGF, FGF, vascular endothelial growth factor and platelet-derived growth factor, thereby promoting cell survival, regeneration and repair (Refs 25, 45).

#### In vivo research of MSC-Exos

Studies have shown that LGMSC-Exos injected into mice through the tail vein (Refs 32, 46) can significantly improve the salivary flow rate and also the number and area of glandular lymphatic infiltration foci. Its therapeutic effect is similar to that of LGMSCs. Ma *et al*. (Ref. 47) found in vitro that UCMSC-Exos reduced the autophagy level of CD4^+^T cells in patients with pSS and also reduced their proliferation and apoptosis. The mechanism of action may be through inhibiting Th17 cell differentiation, inducing Treg cell proliferation, inhibiting the expression of IL-17A, IL-17F (Ref. 47), IFN-*γ*, IL-6, and TNF-*α*, promoting the secretion of TGF-*β* and IL-10 and regulating the imbalance of the Th17/Treg cells. In addition, LGMSC-Exos reduce the expression of PR domain zinc finger protein 1 (PRDM1) by delivering miR125b and inhibiting plasma cell differentiation (Ref. 46), thereby reducing inflammation.

#### In vitro research of MSC-EVs

Currently, there are few preclinical studies of MSC-EVs in SS (Ref. 25). EVs carry proteins and RNA from their parent cells, and the content of MSC-EVs varies with changes in donor cells. Indeed, the therapeutic effect depends largely on the donor, culture conditions and tissue source of MSCs (Ref. 45). Previous studies have shown that the content of EVs varies with changes in the parent cells owing to different culture conditions. Moreover, the biological characteristics of MSCs declined with in vitro amplification, and the effect of late-passage MSC-EVs was lower than that of early-passage MSC-EVs (Ref. 48). Thus, the clinical application of MSCs and their EVs from different tissue sources may be hampered by limited scalability and significant differences in biological characteristics owing to donor and culture conditions. The mechanism of action of MSC-EV needs to be explored in the future to provide a basis for the development of new potential cell-free therapies.

### Engineered MSCs and MSC-Exos

Owing to the large size of MSCs, MSC-Exos easily remain in organs such as the spleen, lung and liver during delivery in vivo or are cleared by the blood circulation; to maintain their biological activity and allow controlled release, they need to be engineered (Ref. 49). Engineering modification methods include genetic, surface and tissue engineering. Genetic engineering includes gene modifications via transfection with viral and non-viral vectors (Ref. 50). Surface modification techniques include enzymatic, chemical and non-covalent modifications. Tissue engineering involves encapsulation in biological materials, among which nanocomposites are the most widely used (Refs 51, 52) and have applications in many areas of biomedicine, such as wound healing, bone and cartilage engineering, heart disease and neurological diseases (Ref. 51).

Some studies have found that MSCs modified by genes can enhance their targeting ability and migration efficiency. However, their application is limited because the genomic integration of viral vectors may increase the possibility of tumour occurrence (Ref. 50).

#### In vivo research of engineered MSCs and MSC-Exos

In recent years, there have been studies using transgenic MSCs expressing human soluble tumour necrosis factor receptor 2 (Ref. 53), MSCs with hypomethylation agent epigenetic modification (Ref. 54), microcycle plasmid construction transfected (Ref. 55) or hydrogels (Ref. 56) to treat RA; the results showed that the engineered MSCs have a stronger effect on the inhibition of arthritis than simple MSCs. MSCs were modified to overexpress IL-37 and then transplanted into lupus mice, which improved lupus-related symptoms and survival rates (Ref. 57). Studies on SLE mainly used lentivirus transfection with MSCs, which improved the symptoms and survival rate of lupus mice (Ref. 57). You *et al*. (Ref. 58) found that after treatment of CIA mice with adipose-derived stem cells, MGE surface modification of MSC-Exos promoted the effective aggregation of engineered Exos in inflamed joints and significantly reduced the inflammatory response of the cartilage and synovial membrane. Moreover, the drug concentration of the engineered MSC-Exos was 10 times lower than that of pure MSC-Exos. These studies revealed that, compared with MSCs or MSC-Exos alone, engineered MSCs or MSC-Exos can better inhibit inflammation, increase drug concentration at the damaged site and have a better therapeutic effect. Although engineered MSCs and MSC-Exos have not been studied for the treatment of SS, this therapy is of great significance as a reference for SS.

### Induced pluripotent stem cells

Somatic cells are differentiated, and mature cells have high specificity. In 2006, Takahashi and Yamanaka (Ref. 59) were the first to use viral vectors to transfer four transcription factors (Oct4, Sox2, Klf4 and c-Myc) to mouse embryos or adult fibroblasts to obtain iPSCs. They are similar to ESCs in expression morphology, growth characteristics and marker genes, and have similar potential for self-renewal and multidirectional differentiation. In addition, they avoid the ethical and immune rejection problems associated with stem cell transplantation. Current studies have confirmed that skin fibroblasts, peripheral blood mononuclear cells (PBMCs), amniotic fluid cells, epidermal keratinocytes, urine cells and other cells can be reprogrammed into iPSCs under specific conditions (Refs 60, 61, 62). iPSCs can be genetically manipulated with high efficiency and reliability. This indicates that an unlimited supply of iPSCs can be generated from a single blood, urine or tissue donor, resulting in large-scale generation of standardised derivatives (Ref. 63).

#### In vivo research of iPSC-MSCs

Currently, MSCs and MSC-EVs derived from iPSCs are mostly used in SS studies. Hai *et al*. (Ref. 64) injected phosphate-buffered saline (PBS), 1 × 10^6^ iPSC-MSCs or BM-MSCs into the caudal vein of NOD mice. The results showed that lymphocyte infiltration in the MSCs group decreased significantly; however, there was no significant difference between the iPSC-MSC and BM-MSC groups. Enzyme-linked immunosorbent assay results showed that the serum levels of anti-La and anti-Ro50 significantly decreased. The B cell marker CD19; B/plasma cell markers CD79a, CD79b, and Ighg3; helper T cell marker CD4; Th17 marker IL17; Treg marker Fxop3; Tfh marker induced T cell co-stimulatory factor (Icos) and its ligand Icosl and activated APCs markers CD40 and IL-10 were detected by quantitative reverse transcription-polymerase chain reaction. The MSC group showed significantly reduced expression of CD19, CD79a, CD79b and Ighg3 in the SMG, whereas the iPSC-MSC group showed more obvious inhibition. The expression of CD4, Fxop3, Icosl and CD40 was downregulated in both the iPSC-MSC and BM-MSC groups, whereas the expression of IL-10 was upregulated, with no difference between the two groups. There was no difference in Icos expression between the MSC and control groups, and IL-17 expression was not detected. In conclusion, iPSC-MSCs were comparable with BM-MSCs. This mechanism may involve exerting immunomodulatory effects by inhibiting T and B cell recruitment and the activation of Tfh and APCs.

#### In vitro and in vivo studies of EVs from iPSC-MSCs

The SGECs of patients with SS express and present autoantigens and provide co-stimulatory molecules, such as CD40, CD80 and CD86, to activate T cells. Moreover, they induced initial CD4^+^ T cell to differentiation into Tfh cells using inducible co-stimulatory molecules (ICOSLG) and IL-6. In a study by Hai *et al*. (Ref. 64), PBMCs were co-cultured with SGECs, and the mRNA levels of Icos, IL-21, IL-12A and IL-17A were upregulated. The mRNA levels of CD40, CD80, CD86, ICOSLG and IFN-*γ* in SGECs were also upregulated. After EV treatment of BM-MSCs and iPSC-MSCs, the above factors were significantly inhibited, but IL-10 mRNA levels in PBMC increased. The mRNA levels of Foxp3 in PBMC and those of IL-6, IL-12A and IL-18 in SGECs were not affected, and ICOSLG mRNA expression was not detected. This suggests that Tregs may not mediate the upregulation of IL-10 by EV but may be caused by the inhibition of Tfh differentiation. However, Tfh differentiation in SGECs was influenced by ICOSLG. These data suggest that EVs from BM-MSCs and iPSC-MSCs can inhibit Tfh and Th17 differentiation induced by SGECs and immune cell interactions, as well as the expression of co-stimulatory molecules and pro-inflammatory factors. In vivo, NOD mice injected with iPSC-MSC-EVs via the tail vein showed significantly decreased lymphocyte infiltration of SMGs, serum anti-La/Ro antibody levels and B-cell-related gene expression. In addition, the inhibitory effect of iPSC-MSC-EVs on the expression of CD3e, CD4, Icos and Icosl and lymphocyte infiltration in the SMG was similar to that of iPSC-MSCs.

#### In vitro and in vivo studies of organoids induced by iPSCs

Since SS can involve multiple systemic systems, such as the lungs, kidneys and also the cardiovascular system, researchers have been using iPSCs to induce various organoids in recent years, which is of great significance for the study of SS organ involvement. Organoids are not human organs in the true sense but three-dimensional (3D) multicellular structures that can approximate real organs in terms of structure and function. They can also simulate the structure and function of tissues in the body to a great extent and are stable for long-term subcultures. Moreover, compared with animal models, they can better reflect human physiology and disease conditions. Currently, 3D organoids induced by iPSCs include lung, kidney, liver and vascular organoids. Human pulmonary organoids (Ref. 65) were generated from hPSCs with bronchi/bronchiole cell types and structures similar to those of human airways. These cells are surrounded by lung mesenchymal cells and cells expressing alveolar cell markers. Various previous studies have shown that the induction of analogue signals (such as Wnt11, Wnt9b and FGF) in vitro helped generate iPSCs renal organoids (Ref. 66). Single-cell RNA sequencing revealed developing podocytes, proximal tubules, distal tubules, collecting tubules and endothelial cells (Ref. 66). Mun *et al*. successfully established iPSCs liver organoids (Ref. 67). They exhibit self-renewal capabilities and functional activities in protein and lipid metabolism, drug metabolism, regeneration and inflammatory responses. Wimmer *et al*. (Ref. 68) prepared vascular organoids induced by iPSC-containing endothelial cells and pericytes that assembled into a network of capillaries wrapped in a basement membrane. When transplanted into mice, a vascular tree with stable perfusion is formed.

Although animal models (most commonly mice) have made significant contributions to the understanding of disease mechanism, they have failed to translate during conversion from experimental animal models to human drug trials (Refs 69, 70). Therefore, iPSCs must be induced into organoids for disease modelling. iPSCs have a considerable impact on medical fields such as cell therapy, disease modelling, drug screening and regenerative medicine. Since iPSCs of different tissue origins retain epigenetic information about the donor tissue origin, they can easily differentiate into the cell types of the donor cell origin (Ref. 61). Therefore, before the actual application of iPSCs in clinical practice, many obstacles need to be solved, such as which tissue cells prepared by iPSCs are the most efficient and can be mass-produced. Only in this manner can the real potential of iPSCs be effectively translated into clinical practice to better serve patients.

### Salivary gland stem cells

SGSCs can self-renew and differentiate into specific cell types (Ref. 71). Patients with SS or undergoing radiotherapy of the head and neck have symptoms of dry mouth, and their salivary gland function cannot recover. Although artificial saliva substitutes, salivary tubes and systemic parasympathetic pathological mimics can be used to treat these conditions, these are all palliative approaches. Mouse ESCs can be gradually induced to develop into early salivary glands and mature further after *in situ* transplantation, demonstrating the feasibility of using functional salivary glands instead of organs. However, this approach is limited by the tumourigenicity of mouse ESCs and the heterogeneity of animal-derived cells. Human adult stem cells have been used as alternatives to construct organoids with specific structures and functions.

#### In vivo research of SGSCs

In one study by Sui *et al*. (Ref. 71) isolated human submaxillary gland stem/progenitor cells (hSMGepiS/PCs) for 3D culture to generate organoids that were further induced by FGF-10 in vitro. hSMGepiS/PC-derived spheres were transplanted into the renal capsule of nude mice alone or in combination with the salivary gland mesenchyma of mice on the 12th day of the embryo. hSMGepiS/PC-derived spheres reacted with the embryonic salivary gland mesenchyma of mice and developed into salivary glands with the correct structure and independent secretory function in vivo. This not only confirmed the regenerative potential of hSMGepiS/PCs but also showed that hSMGepiS/PCs responded to the mouse embryonic mesenchymal niche and further differentiated. Jeong *et al*. (Ref. 72) isolated and cultured tissue-specific stem cells, namely human salivary gland stem cells (hSGSCs), which express stem cell surface antigen markers such as CD44, CD49f, CD90 and CD105, but not HSC markers such as CD34 and CD45. Next, hSGSCs or PBS were injected intravenously into Wistar rats with radiation-induced damage. The body weight of the rats in the hSGSCs group increased significantly compared with that in the PBS group; the salivary flow rate increased two-fold, and the structure of salivary glands showed a compact acicular structure similar to that of the undamaged normal rats, suggesting that hSGSCs can improve the structure and function of rat salivary glands and may have the characteristics of stem cells, which can be used as a cell therapy agent for damaged salivary glands.

#### In vitro research of SGSCs

Partial functional recovery of the parotid gland (PG) after a certain threshold of radiation therapy indicates the presence of stem or progenitor cells that proliferate and differentiate, allowing for tissue regeneration in response to injury. Serrano Martinez *et al*. (Ref. 73) isolated PG stem/progenitor cells from the PGs of female C57BL/6 mice and observed elevated expression of CK14 (basal transverse duct marker of the PG), AQP5 (acinar cell marker), CK8 (luminal PG duct marker) and amylase after 3D culture. This indicates that the ability to self-renew, differentiate and expand over a long period supports the stem cell or organoid characteristics. According to in vitro the 3D cultures of stem/progenitor cells, they possess stem cell characteristics. Thus, they can provide a new method for the treatment of SS, promote the development of organoids, provide a basis for subsequent organoid transplantation, disease modelling and drug screening, and lay the foundation for clinical transformation.

## Immune cell therapy

In addition to the aforementioned stem cell therapy, research on immune cell therapy for autoimmune diseases continues to emerge. Immunocell therapy includes chimeric antigen receptor T cell (CAR-T) and adoptive cell therapy. Currently, CAR-T cells are the most studied cells in the field of autoimmune diseases (Refs 74, 75). CAR-T therapy involves the in vitro activation of T cells collected from the peripheral blood of patients to induce the expression of chimeric antigen receptors and their expansion. The modified CAR-T is transfused back into the patient's body to specifically identify and bind the corresponding antigen such that CAR-T cells can activate, proliferate, and kill target cells and continue to restore immune balance. Currently, the strategy for studying autoimmune diseases focuses on CAR-CD8^+^T cell therapy, CAR-CD4^+^T cell therapy, and CAR-Treg cell therapy.

### In vivo research of CAR-T

At present, there are only a few related studies on SS and many reports on SLE. In studies by Kansal *et al*. (Ref. 76) and Jin *et al*. (Ref. 77) on CAR-T therapy for SLE, CAR-T cells targeting CD19^+^B cells were injected into MRL-lpr lupus mice, and the results showed that the survival time of the CAR-T group was prolonged, in the spleen, bone marrow, and blood were not detected CD19^+^B cells. Jin *et al*. (Ref. 77) found that skin symptoms and kidney involvement improved in MRL-lpr lupus mice injected with CAR-T cells before disease onset. Thus, CAR-T therapy can prevent or ameliorate disease, prolong the lifespan of mice, and continue to consume CD19^+^B cells. In a clinical study, a patient with severe and refractory SLE patient (Ref. 78) who was treated with hydroxychloroquine, high-dose glucocorticoids, cyclophosphamide, B-cell targeting drugs, and other treatments was not controlled. After CAR-T cell therapy, circulating B cells continued to be completely consumed; C3 and C4 levels increased or even normalised; and anti-dsDNA antibody levels, urinary protein levels, and SLE disease activity index scores decreased. In a clinical trial conducted by Mackensen *et al*. (Ref. 79), a total of five patients with SLE were recruited. After receiving CAR-CD4^+^T therapy targeting CD19^+^B cells, clinical symptoms improved, B cells in the peripheral blood disappeared, CAR-T cell proliferation, and B cell reconstruction were observed, and no traditional drug therapy was required during long-term follow-up. Based on these data, CAR-T therapy targeting CD19^+^B cells can sustain the deep clearance of B cells in patients with SLE, and the therapeutic effect persists even after B cell reconstruction without serious side effects. Thus, CAR-T cells are primarily used to treat SLE by targeting D19^+^B cells, resulting in the complete and sustained depletion of B cells that produce autoimmune antibodies. A study on CD19/BCMA CAR-Ts for the treatment of patients with refractory SS retrieved from the Clinical Trials website is still underway, and the results have not been published yet. Despite limited clinical data, the results of CAR-T cell therapy in SLE may become a new scheme for treating SS in the future.

### In vivo research of CAR-Treg

Treg cells (Refs 80, 81, 82, 83, 84) are a type of T cells, accounting for 5–10% of CD4^+^T cells. Treg cells play an important role in maintaining homoeostasis and regulating autoimmunity, as well as promoting tissue repair and regulating metabolism, which suppress the immune response through various mechanisms, including direct interaction with other immune cells or the production of immunosuppressive cytokines, such as IL-10, IL-35 and TGF-*β*. At present, Treg cell therapy has not been studied in SS but has been reported in multiple sclerosis. Fransson *et al*. (Ref. 85) investigated the therapeutic effect of Treg cells on multiple sclerosis by creating antigen-specific Tregs targeting myelin oligodendrocyte glycoprotein (MOG) with CAR and injecting them into experimental autoimmune encephalomyelitis (EAE) models with MS. They found that MOG–CAR–Tregs inhibit the proliferation of effector T cells in vitro. Meanwhile, MOG–CAR–Treg reduced the disease symptoms in EAE mice and reduced the levels of pro-inflammatory cytokines (IL-12, INF-*γ*) in brain tissue in vivo.

Although CAR-T cells have been poorly studied in SS, according to the findings in SLE and multiple sclerosis, they can be used as a novel treatment for SS. However, owing to limited clinical data, conducting long-term follow-ups in large clinical trials is imperative to clarify the safety and efficacy, as well as provide a basis for CAR-T therapy of SS and hope for more patients.

## Conclusions

For SS cell therapy, HSCs have strong proliferation and differentiation abilities in the primitive stage, and their self-renewal is rapid. MSCs exhibit high proliferation, multidirectional differentiation and immunomodulatory abilities. Small MSC-EVs can quickly pass through capillaries, have stable properties and do not decline over time. The homing ability of the engineered MSCs and MSC-Exos was strong, and their therapeutic effects were optimal. iPSCs have the same potential for self-renewal and multi-differentiation as ESCs while avoiding the ethical and immune rejection problems of ESCs. They can also induce the formation of organoids, such as lungs, kidneys, blood vessels and salivary glands, which have great potential in the construction of disease models, screening of drugs, the study of pathogenesis, treatment and regeneration, and have great potential application value. Preclinical data have demonstrated the safety of CAR-T-cell therapy. However, HSCs differ in terms of stem cell collection, pretreatment, and GVHD after transplantation. MSCs are limited in terms of tissue origin heterogeneity, cell preparation, number of target organs reached and survival time (Ref. 64). However, many problems still need to be solved before MSC-EVs can be used clinically. The culture conditions, mass production, rapid and accurate quantification, characterisation methods, pharmacokinetics and determination of the optimal clinical dose must be improved (Ref. 86). Engineered MSCs and MSC-Exos present difficulties in their preparation, composite side effects and transfection efficiency. iPSCs must solve the problems of donor cells or tissues, low reprogramming efficiency, carrier selection and transcription factor combinations. There are many obstacles to the preparation, mass production and translation of organoids. CAR-T cell therapy must address cell acquisition, gene introduction, amplification, mass production, preservation methods and quality detection (Ref. 87).

Different cell therapy methods have been shown to have different advantages, and appropriate cell therapy should be chosen according to different needs to better utilise different cells in the treatment of diseases.

## References

[1] Mariette X and Criswell LA (2018) Primary Sjogren's syndrome. The New England Journal of Medicine 378, 931–939. 29514034 10.1056/NEJMcp1702514

[2] Negrini S et al. (2022) Sjogren's syndrome: a systemic autoimmune disease. Clinical and Experimental Medicine 22, 9–25. 34100160 10.1007/s10238-021-00728-6 PMC8863725

[3] Xu D et al. (2020) Characteristics of Chinese patients with primary Sjögren's syndrome: preliminary report of a multi-centre registration study. Lupus Science Medicine 29, 45–51. 10.1177/0961203319889666 31793380

[4] Retamozo S et al. (2019) Prognostic markers of lymphoma development in primary Sjögren syndrome. Lupus Science Medicine 28, 923–936. 10.1177/0961203319857132 31215845

[5] Sandhya P et al. (2017) Update on pathogenesis of Sjogren's syndrome. Current Rheumatology Reports 13, 5–22. 10.2174/1573397112666160714164149 PMC5280579 27412602

[6] An Q et al. (2022) Exploiting the role of T cells in the pathogenesis of Sjögren's syndrome for therapeutic treatment. Frontiers in Immunology 13, 995895. 36389806 10.3389/fimmu.2022.995895 PMC9650646

[7] Srivastava A and Makarenkova HP (2020) Innate immunity and biological therapies for the treatment of Sjogren's syndrome. International Journal of Molecular Sciences 21, 9172. 33271951 10.3390/ijms21239172 PMC7730146

[8] Maslinska M and Kostyra-Grabczak K (2022) The role of virus infections in Sjögren's syndrome. Frontiers in Immunology 13, 823659. 36148238 10.3389/fimmu.2022.823659 PMC9488556

[9] Ramos-Casals M et al. (2020) EULAR recommendations for the management of Sjogren's syndrome with topical and systemic therapies. Annals of the Rheumatic Diseases 79, 3–18. 31672775 10.1136/annrheumdis-2019-216114

[10] de Wolff L et al. (2022) Long-term abatacept treatment for 48 weeks in patients with primary Sjögren's syndrome: the open-label extension phase of the ASAP-III trial. Seminars in Arthritis and Rheumatism 53, 151955. 35091325 10.1016/j.semarthrit.2022.151955

[11] Tan Z et al. (2022) Composition and regulation of the immune microenvironment of salivary gland in Sjögren's syndrome. Frontiers in Immunology 13, 967304. 36177010 10.3389/fimmu.2022.967304 PMC9513852

[12] Marinho A et al. (2023) Biological therapy in systemic lupus erythematosus, antiphospholipid syndrome, and Sjögren's syndrome: evidence- and practice-based guidance. Frontiers in Immunology 14, 1117699. 37138867 10.3389/fimmu.2023.1117699 PMC10150407

[13] Shihai Z and Ping L (2022) Research progress in the treatment of primary Sjogren's syndrome with biological agents. Chinese Journal of Modern Applied Pharmacy 39, 2293–2300.

[14] Ridgewell D et al. (2022) Sjögren's syndrome: shedding light on emerging and key drug targets. Expert Opinion on Therapeutic Targets 26, 869–882. 36576336 10.1080/14728222.2022.2157259

[15] El-Kadiry AE et al. (2021) Cell therapy: types, regulation, and clinical benefits. Frontiers in Medicine 8, 756029. 34881261 10.3389/fmed.2021.756029 PMC8645794

[16] Biehl JK and Russell B (2009) Introduction to stem cell therapy. The Journal of Cardiovascular Nursing 24, 98–103. 19242274 10.1097/JCN.0b013e318197a6a5 PMC4104807

[17] Balassa K et al. (2019) Haematopoietic stem cell transplants: principles and indications. British Journal of Hospital Medicine (London, England: 2005) 80, 33–39. 30592675 10.12968/hmed.2019.80.1.33

[18] Lutter L et al. (2018) Resetting the T cell compartment in autoimmune diseases with autologous hematopoietic stem cell transplantation: an update. Frontiers in Immunology 9, 767. 29731752 10.3389/fimmu.2018.00767 PMC5920130

[19] Choi HN et al. (2013) Remission of lymphocytic interstitial pneumonia in Sjögren's syndrome after autologous peripheral blood stem cell transplantation. Journal of Rheumatic Diseases 20, 118–122.

[20] Isshiki I et al. (2006) Recurrence of autoimmune disease after autologous peripheral blood stem cell transplantation for multiple myeloma. International Journal of Hematology 84, 354–358. 17118763 10.1532/IJH97.06029

[21] Kaneko H et al. (2006) Simultaneous complication of multiple myeloma with Sjögren syndrome. Asian Pacific Journal of Allergy and Immunology 24, 245–248. 17348248

[22] Ferraccioli G et al. (2001) Haematopoietic stem cell transplantation (HSCT) in a patient with Sjögren's syndrome and lung MALT lymphoma cured lymphoma not the autoimmune disease. Annals of the Rheumatic Diseases 60, 174–176. 10.1136/ard.60.2.173b PMC1753470 11203719

[23] Rösler W et al. (1998) Autologous PBPCT in a patient with lymphoma and Sjögren's syndrome: complete remission of lymphoma without control of the autoimmune disease. Bone Marrow Transplantation 22, 211–213. 9707034 10.1038/sj.bmt.1701312

[24] Chen W et al. (2018) Mesenchymal stem cells in primary Sjögren's syndrome: prospective and challenges. Stem Cells International 2018, 4357865. 30305818 10.1155/2018/4357865 PMC6165618

[25] Dan M et al. (2020) Research progress of mesenchymal stem cells and their extracellular vesicles in the treatment of Sjögren's syndrome. Chinese Journal of Rheumatology 24, 634–637.

[26] Xu J et al. (2012) Allogeneic mesenchymal stem cell treatment alleviates experimental and clinical Sjögren syndrome. Blood 120, 3142–3151. 22927248 10.1182/blood-2011-11-391144 PMC3471521

[27] Cong Y et al. (2022) Umbilical cord mesenchymal stem cells alleviate Sjögren's syndrome and related pulmonary inflammation through regulating V*γ*4(+) IL-17(+) T cells. Annals of Translational Medicine 10, 594. 35722394 10.21037/atm-22-1855 PMC9201169

[28] Lu X et al. (2020) Human umbilical cord mesenchymal stem cells alleviate ongoing autoimmune dacryoadenitis in rabbits via polarizing macrophages into an anti-inflammatory phenotype. Experimental Eye Research 191, 107905. 31891674 10.1016/j.exer.2019.107905 PMC8612174

[29] Liu Y et al. (2021) Human umbilical cord mesenchymal stem cells confer potent immunosuppressive effects in Sjögren's syndrome by inducing regulatory T cells. Modern Rheumatology 31, 186–196. 31859545 10.1080/14397595.2019.1707996

[30] Shi B et al. (2018) Mesenchymal stem cell transplantation ameliorates Sjögren's syndrome via suppressing IL-12 production by dendritic cells. Stem Cell Research & Therapy 9, 308. 30409219 10.1186/s13287-018-1023-x PMC6225717

[31] Yao G et al. (2019) Mesenchymal stem cell transplantation alleviates experimental Sjögren's syndrome through IFN-*β*/IL-27 signaling axis. Theranostics 9, 8253–8265. 31754394 10.7150/thno.37351 PMC6857067

[32] Li B et al. (2021) Labial gland-derived mesenchymal stem cells and their exosomes ameliorate murine Sjögren's syndrome by modulating the balance of Treg and Th17 cells. Stem Cell Research & Therapy 12, 478. 34446113 10.1186/s13287-021-02541-0 PMC8390194

[33] Liang J et al. (2018) Safety analysis in patients with autoimmune disease receiving allogeneic mesenchymal stem cells infusion: a long-term retrospective study. Stem Cell Research & Therapy 9, 312. 30428931 10.1186/s13287-018-1053-4 PMC6236873

[34] Yoo C et al. (2014) Adult stem cells and tissue engineering strategies for salivary gland regeneration: a review. Biomaterials Research 18, 9. 26331060 10.1186/2055-7124-18-9 PMC4549133

[35] Khalili S et al. (2012) Mesenchymal stromal cells improve salivary function and reduce lymphocytic infiltrates in mice with Sjögren's-like disease. Public Library of Science 7, e38615. 10.1371/journal.pone.0038615 PMC3369846 22685592

[36] Sun T et al. (2022) Mesenchymal stem cell transplantation alleviates Sjögren's syndrome symptoms by modulating Tim-3 expression. International Immunopharmacology 111, 109152. 36007392 10.1016/j.intimp.2022.109152

[37] Yamanaka S (2020) Pluripotent stem cell-based cell therapy-promise and challenges. Cell Stem Cell 27, 523–531. 33007237 10.1016/j.stem.2020.09.014

[38] Wang S et al. (2015) Excess integrins cause lung entrapment of mesenchymal stem cells. Stem Cells (Dayton, Ohio) 33, 3315–3326. 26148841 10.1002/stem.2087

[39] Wang S et al. (2018) Measurement of mesenchymal stem cells attachment to endothelial cells. Bio-Protocol 8, e2776. 34179290 10.21769/BioProtoc.2776 PMC8203963

[40] Fennema EM et al. (2018) Ectopic bone formation by aggregated mesenchymal stem cells from bone marrow and adipose tissue: a comparative study. Journal of Tissue Engineering and Regenerative Medicine 12, e150–e158. 28485099 10.1002/term.2453

[41] Huang Y et al. (2020) Recent advances in the use of exosomes in Sjögren's syndrome. Frontiers in Immunology 11, 1509. 32903777 10.3389/fimmu.2020.01509 PMC7438915

[42] Liu H et al. (2020) Immunomodulatory effects of mesenchymal stem cells and mesenchymal stem cell-derived extracellular vesicles in rheumatoid arthritis. Frontiers in Immunology 11, 1912. 32973792 10.3389/fimmu.2020.01912 PMC7468450

[43] Zeng G et al. (2021) Application of extracellular vesicles derived from mesenchymal stem cells in body repair and disease therapy. Science China Life Sciences 51, 1229–1240.

[44] Zhao J et al. (2022) Research status and future prospects of extracellular vesicles in primary Sjögren's syndrome. Stem Cell Research & Therapy 13, 230. 35659085 10.1186/s13287-022-02912-1 PMC9166483

[45] Kim H et al. (2020) Comprehensive molecular profiles of functionally effective MSC-derived extracellular vesicles in immunomodulation. Molecular Therapy: The Journal of the American Society of Gene Therapy 28, 1628–1644. 32380062 10.1016/j.ymthe.2020.04.020 PMC7335740

[46] Xing Y et al. (2022) Labial gland mesenchymal stem cell derived exosomes-mediated miRNA-125b attenuates experimental Sjogren's syndrome by targeting PRDM1 and suppressing plasma cells. Frontiers in Immunology 13, 871096. 35444638 10.3389/fimmu.2022.871096 PMC9014006

[47] Ma D et al. (2023) Immunomodulatory effects of umbilical mesenchymal stem cell-derived exosomes on CD4(+) T cells in patients with primary Sjögren's syndrome. Inflammopharmacology 31, 1823–1838. 37012581 10.1007/s10787-023-01189-x PMC10352432

[48] Kim H et al. (2021) Identification of molecules responsible for therapeutic effects of extracellular vesicles produced from iPSC-derived MSCs on Sjogren's syndrome. Aging and Disease 12, 1409–1422. 34527418 10.14336/AD.2021.0621 PMC8407887

[49] Zhu X et al. (2023) Research progress of engineered mesenchymal stem cells and their derived exosomes and their application in autoimmune/inflammatory diseases. Stem Cell Research & Therapy 14, 71. 37038221 10.1186/s13287-023-03295-7 PMC10088151

[50] Shin MJ et al. (2021) Stem cell mimicking nanoencapsulation for targeting arthritis. International Journal of Nanomedicine 16, 8485–8507. 35002240 10.2147/IJN.S334298 PMC8725870

[51] Raghav PK et al. (2022) Mesenchymal stem cell-based nanoparticles and scaffolds in regenerative medicine. European Journal of Pharmacology 918, 174657. 34871557 10.1016/j.ejphar.2021.174657

[52] Khayambashi P et al. (2021) Hydrogel encapsulation of mesenchymal stem cells and their derived exosomes for tissue engineering. International Journal of Molecular Sciences 22, 684. 33445616 10.3390/ijms22020684 PMC7827932

[53] Liu LN et al. (2013) Comparison of drug and cell-based delivery: engineered adult mesenchymal stem cells expressing soluble tumor necrosis factor receptor II prevent arthritis in mouse and rat animal models. Stem Cells Translational Medicine 2, 362–375. 23592838 10.5966/sctm.2012-0135 PMC3667563

[54] Kim KW et al. (2018) Epigenetic modification of mesenchymal stromal cells enhances their suppressive effects on the Th17 responses of cells from rheumatoid arthritis patients. Stem Cell Research & Therapy 9, 208. 30092847 10.1186/s13287-018-0948-4 PMC6085688

[55] Park N et al. (2017) Etanercept-synthesising mesenchymal stem cells efficiently ameliorate collagen-induced arthritis. Scientific Reports 7, 39593. 28084468 10.1038/srep39593 PMC5234034

[56] Gang F et al. (2021) Thermochemotherapy meets tissue engineering for rheumatoid arthritis treatment. Advanced Functional Materials 31, 2104131.

[57] Xu J et al. (2020) Additive therapeutic effects of mesenchymal stem cells and IL-37 for systemic lupus erythematosus. Journal of the American Society of Nephrology: JASN 31, 54–65. 31604808 10.1681/ASN.2019050545 PMC6935004

[58] You DG et al. (2021) Metabolically engineered stem cell-derived exosomes to regulate macrophage heterogeneity in rheumatoid arthritis. Science Advances 7, eabe0083. 34078596 10.1126/sciadv.abe0083 PMC8172131

[59] Takahashi K and Yamanaka S (2006) Induction of pluripotent stem cells from mouse embryonic and adult fibroblast cultures by defined factors. Cell 126, 663–676. 16904174 10.1016/j.cell.2006.07.024

[60] Takahashi K et al. (2007) Induction of pluripotent stem cells from adult human fibroblasts by defined factors. Cell 131, 861–872. 18035408 10.1016/j.cell.2007.11.019

[61] Yong F et al. (2012) Studies on the establishment of induced pluripotent stem cell lines from cells of different tissue origin. Progress in Modern Biomedicine 12, 5621–5625.

[62] Gautam S et al. (2021) Urine cells-derived iPSCs: an upcoming frontier in regenerative medicine. Current Medicinal Chemistry 28, 6484–6505. 34165400 10.2174/0929867328666210623142150

[63] Bertolino GM et al. (2022) Recent advances in extracellular vesicle-based therapies using induced pluripotent stem cell-derived mesenchymal stromal cells. Biomedicines 10, 2281. 36140386 10.3390/biomedicines10092281 PMC9496279

[64] Hai B et al. (2018) Inhibitory effects of iPSC-MSCs and their extracellular vesicles on the onset of sialadenitis in a mouse model of Sjögren's syndrome. Stem Cells International 2018, 2092315. 29736173 10.1155/2018/2092315 PMC5875028

[65] Miller AJ et al. (2019) Generation of lung organoids from human pluripotent stem cells *in vitro*. Nature Protocols 14, 518–540. 30664680 10.1038/s41596-018-0104-8 PMC6531049

[66] Yousef Yengej FA et al. (2020) Kidney organoids and tubuloids. Cells 9, 1326. 32466429 10.3390/cells9061326 PMC7349753

[67] Mun SJ et al. (2019) Generation of expandable human pluripotent stem cell-derived hepatocyte-like liver organoids. Journal of Hepatology 71, 970–985. 31299272 10.1016/j.jhep.2019.06.030

[68] Wimmer RA et al. (2019) Human blood vessel organoids as a model of diabetic vasculopathy. Nature 565, 505–510. 30651639 10.1038/s41586-018-0858-8 PMC7116578

[69] Ohnuki M and Takahashi K (2015) Present and future challenges of induced pluripotent stem cells. Philosophical Transactions of the Royal Society of London. Series B, Biological Sciences 370, 20140367. 26416678 10.1098/rstb.2014.0367 PMC4633996

[70] Doss MX and Sachinidis A (2019) Current challenges of iPSC-based disease modeling and therapeutic implications. Cells 8, 403. 31052294 10.3390/cells8050403 PMC6562607

[71] Sui Y et al. (2020) Generation of functional salivary gland tissue from human submandibular gland stem/progenitor cells. Stem cell Research & Therapy 11, 127. 32197647 10.1186/s13287-020-01628-4 PMC7083056

[72] Jeong J et al. (2013) Human salivary gland stem cells ameliorate hyposalivation of radiation-damaged rat salivary glands. Experimental & Molecular Medicine 45, e58. 24232257 10.1038/emm.2013.121 PMC3849572

[73] Serrano Martinez P et al. (2021) Mouse parotid salivary gland organoids for the *in vitro* study of stem cell radiation response. Oral Diseases 27, 52–63. 32531849 10.1111/odi.13475 PMC7818507

[74] Pengcheng W and Zhihong L (2019) Research progress of chimeric antigen receptor T cell immunotherapy in autoimmune diseases. Chinese Journal of Nephrology Dialysis & Transplantation 28, 550–555.

[75] Zmievskaya E et al. (2021) Application of CAR-T cell therapy beyond oncology: autoimmune diseases and viral infections. Biomedicines 9, 59. 33435454 10.3390/biomedicines9010059 PMC7827151

[76] Kansal R et al. (2019) Sustained B cell depletion by CD19-targeted CAR T cells is a highly effective treatment for murine lupus. Science Translational Medicine 11, eaav1648. 30842314 10.1126/scitranslmed.aav1648 PMC8201923

[77] Jin X et al. (2021) Therapeutic efficacy of anti-CD19 CAR-T cells in a mouse model of systemic lupus erythematosus. Cellular & Molecular Immunology 18, 1896–1903. 32472023 10.1038/s41423-020-0472-1 PMC8322088

[78] Mougiakakos D et al. (2021) CD19-Targeted CAR T cells in refractory systemic lupus erythematosus. The New England Journal of Medicine 385, 567–569. 34347960 10.1056/NEJMc2107725

[79] Mackensen A et al. (2022) Anti-CD19 CAR T cell therapy for refractory systemic lupus erythematosus. Nature Medicine 28, 2124–2132. 10.1038/s41591-022-02017-5 36109639

[80] Riet T and Chmielewski M (2022) Regulatory CAR-T cells in autoimmune diseases: progress and current challenges. Frontiers in Immunology 13, 934343. 36032080 10.3389/fimmu.2022.934343 PMC9399761

[81] Mohseni YR et al. (2020) The future of regulatory T cell therapy: promises and challenges of implementing CAR technology. Frontiers in Immunology 11, 1608. 32793236 10.3389/fimmu.2020.01608 PMC7393941

[82] Arjomandnejad M et al. (2022) CAR-T regulatory (CAR-Treg) cells: engineering and applications. Biomedicines 10, 287. 35203496 10.3390/biomedicines10020287 PMC8869296

[83] Ferreira LMR et al. (2019) Next-generation regulatory T cell therapy. Nature Reviews Drug Discovery 18, 749–769. 31541224 10.1038/s41573-019-0041-4 PMC7773144

[84] Zhuobei X et al. (2022) Research progress of regulatory T cells in the pathogenesis of systemic lupus erythematosus. Cellular & Molecular Immunology 38, 565–570.

[85] Fransson M et al. (2012) CAR/FoxP3-engineered T regulatory cells target the CNS and suppress EAE upon intranasal delivery. Journal of Neuroinflammation 9, 112. 22647574 10.1186/1742-2094-9-112 PMC3403996

[86] Moghadasi S et al. (2021) A paradigm shift in cell-free approach: the emerging role of MSCs-derived exosomes in regenerative medicine. Journal of Translational Medicine 19, 302. 34253242 10.1186/s12967-021-02980-6 PMC8273572

[87] Fritsche E et al. (2020) Toward an optimized process for clinical manufacturing of CAR-Treg cell therapy. Trends in Biotechnology 38, 1099–1112. 31982150 10.1016/j.tibtech.2019.12.009

